# A framework for the acoustic simulation of passing vehicles using variable length delay lines

**DOI:** 10.1186/s13636-024-00372-4

**Published:** 2024-10-03

**Authors:** Stefano Damiano, Luca Bondi, Andre Guntoro, Toon van Waterschoot

**Affiliations:** 1 Department of Electrical Engineering (ESAT), STADIUS Center for Dynamical Systems, Signal Processing and Data Analytics, KU Leuven, Kasteelpark Arenberg 10, Leuven, 3001 Belgium; 2 Robert Bosch LLC, 2555 Smallman St, Pittsburgh, PA 15222 USA; 3 Robert Bosch GmbH, Robert-Bosch-Campus 1, Renningen, 71282 Germany

**Keywords:** Acoustic simulation, Traffic noise synthesis, Variable length delay lines, Road acoustic propagation, Doppler effect

## Abstract

The sound produced by vehicles driving on roadways constitutes one of the dominant noise sources in urban areas. The impact of traffic noise on human activities and the related investigation on modeling, assessment, and abatement strategies fueled the research on the simulation of the sound produced by individual passing vehicles. Simulators enable in fact to promote a perceptual assessment of the nature of traffic noise and of the impact of single road agents on the overall soundscape. In this work, we present *TrafficSoundSim*, an open-source framework for the acoustic simulation of vehicles transiting on a road. We first discuss the generation of the sound signal produced by a vehicle, represented as a combination of road/tire interaction noise and engine noise. We then introduce a propagation model based on the use of variable length delay lines, allowing to simulate acoustic propagation and Doppler effect. The proposed simulator incorporates the effect of air absorption and ground reflection, modeled via complex-valued reflection coefficients dependent on the road surface impedance, as well as a model of the directivity of sound sources representing the passing vehicles. The source signal generation and the propagation stages are decoupled, and all effects are implemented using finite impulse response filters. Moreover, no recorded data is required to run the simulation, making the framework flexible and independent on data availability. Finally, to validate the framework capability to accurately simulate passing vehicles, a comparison between synthetic and recorded pass-by events is presented. The validation shows that sounds generated with the proposed method achieve a good match with recorded events in terms of power spectral density and psychoacoustics metrics as well as a perceptually plausible result.

**Supplementary Information:**

The online version contains supplementary material available at 10.1186/s13636-024-00372-4.

## Introduction

The noise produced by public and private transportation systems is a prominent feature of the urban soundscape. In any major city, vehicles such as cars, trucks, trains, and aircraft are in fact the source of an incessant background noise that, on one hand, has a major impact on the health and quality of life of people [1] and on the other is a source of information on events happening in urban areas [2]. To improve the livability of cities, several noise abatement techniques have been proposed. Since road traffic constitutes the prominent noise source, solutions to mitigate it have been specifically designed via the production of quieter engines and the manufacture of tires and asphalt mixtures designed to limit the rolling sound. Simultaneously, urban studies aiming at diverting the traffic flow to reduce vehicle density in urban areas, as well as the installation of sound barriers, have proven effective to reduce the overall sound intensity [1, 3].

To ease the study and assessment of traffic noise, engineering methods that mostly aim at the evaluation of average sound pressure levels (SPL) [4, 5] have been developed. While effective for estimating the long-term averaged acoustic pressure, such tools are usually insufficient to capture the complexity of the highly non-stationary urban soundscape, including individual vehicle pass-bys, impulsive noises, and emergency sounds such as horns and sirens.

For this reason, auralization techniques have been exploited to provide the opportunity to *listen* to the sound that would be received by a listener in a specific location [2, 3, 6–9]. According to the definition provided in [10], auralization refers to the technique of creating audible sound from numeric data. The auralization of a virtual environment involves three main steps: i) the definition of a virtual acoustic scene containing a set of sound sources and a listener; ii) the simulation of sound propagation given the defined scene and an acoustic propagation model; iii) (optionally) the spatial audio rendering to reproduce the sound perceived by the listener (i.e., binaural rendering). Although auralization techniques are largely exploited in indoor acoustics studies and in the early phases of the design of closed spaces (e.g., concert halls, recording studios, public spaces), the lack of well-established techniques for the auralization of outdoor environments poses a limitation to its adoption for the analysis of urban areas and the assessment of traffic noise. In recent years, many studies have proposed methodologies to implement the different components involved in the auralization of traffic noise, from the definition of source signals to emulate passing vehicles [2, 3, 6, 7, 11–13], to the development of models for acoustic propagation on a road [3, 12, 14–16], to the design of a spatial audio reproduction setup [2, 14, 17].

Auralization tools can be useful both to reproduce the urban acoustic soundscape and to extract some information that it contains. The analysis of traffic noise, in particular, has been exploited in the design of algorithms to enhance the perceptual abilities of autonomous vehicles by means of acoustic sensing [18] and in acoustic vehicle counting systems [19, 20]. In these use-cases, auralization tools can become useful to generate data to train data-driven techniques [20]. The reproduction of traffic soundscapes, instead, has found applications in virtual reality and computer games [2].

With the goal of developing a traffic sound auralization tool, in our previous work [16], we introduced an open-source simulator of acoustic propagation on a road based on the use of variable length delay lines. A delay line allows to effectively simulate the sound produced by a moving source with arbitrary trajectory and speed as well as the Doppler effect, particularly relevant in road scenarios due to the relatively high speed that vehicles can reach. The simulator incorporates a model of air absorption and asphalt reflection, implemented via finite impulse response (FIR) filters, and supports user-defined source signals, trajectories, and speeds. Compared to more complex propagation models relying on wave-based methods, *pyroadacoustics* is based on geometrical acoustics and comes with a lower computational complexity.

This work extends [16] by introducing *TrafficSoundSim*, a framework for the acoustic simulation of passing vehicles. *TrafficSoundSim* is composed of two independent modules: the source synthesis module, responsible for the generation of source signals to represent vehicular sources, and the propagation module, responsible for the simulation of acoustic propagation on a road. For the design of the source synthesis module, we adopt a sound source description inspired by the Harmonoise model [21], according to which the sound produced by road vehicles can be split into two main contributions, namely road/tire interaction (or rolling) sound and engine (or propulsion) sound. Every road vehicle can then be acoustically described by two vertically stacked point sources, emitting a mixture of rolling and propulsion sound, both broadband in nature.

In Harmonoise, the sound produced by a passing vehicle is synthesized in short-time windows, within which the source is assumed to be static and propagation effects are simulated jointly with the signal generation. This model comes with two main drawbacks. First, while effective for characterizing road/tire interaction noise, the broadband noise description disregards both the *texture* of a real engine sound, having rumbling and rattling components, as well as prominent tonal characteristics connected to the engine load and speed, and to the speed of the vehicle.

To overcome this limitation, similarly to [2], we introduce a more accurate engine sound generation procedure based on physical modeling, known as the Baldan model [11], on top the Harmonoise method. Both the original Harmonoise and the extendend Harmonoise and Baldan source models have been implemented in the simulator, and a choice between them can be made based on psychoacoustics considerations and on the target application. Second, the joint source signal and acoustic propagation synthesis that is performed within Harmonoise inevitably leads to artifacts, caused by the source *jumping* between successive discrete positions corresponding to the short-time windows used in the synthesis process. Moreover, the simulation of a signal received by multiple receivers entails performing the entire simulation from scratch per each receiver. *TrafficSoundSim*, on the contrary, is designed with a modular structure to keep the source synthesis and propagation modules decoupled. On the one hand, this allows to generate the stationary source signal once and to apply the propagation effects multiple times only in presence of multiple microphones. On the other hand, the use of delay lines reduces the artifacts as the source position is updated sample by sample.

As an additional contribution, we extend the *pyroadacoustics* propagation module: first, we introduce an improved implementation of the ground reflection filtering inspired by the Delany Bazley model [22]. Finally, to simulate the directivity of moving sound sources representing passing vehicles, we introduce an FIR implementation of the Harmonoise directivity function. Differently from the original Harmonoise model, where directivity is implemented at the source signal generation time, the proposed implementation is part of the propagation module, as it represents an effect caused by the movement of the source. This way, the modularity principle of the proposed framework is respected.

The rest of the paper is organized as follows. In Section 2, we discuss the existing literature on the auralization of passing vehicles, with a focus on the Harmonoise and Baldan models that constitute the backbone of *TrafficSoundSim*. In Section 3, we introduce the passing vehicles simulation framework by describing the source synthesis module (Section 3.1), the propagation module (Section 3.2), and the complete simulation setup (Section 3.3). We then show how simulations compare to real pass-by recordings to discuss the effectiveness of the framework in Section 4, and we finally draw conclusions in Section 5.

## Related work

The existing frameworks for the auralization of vehicle pass-by events consist of three main building blocks, responsible for the generation of the source signal, the propagation of sound from the source to the receiver, and (optionally) the spatial sound reproduction (i.e., binaural rendering). Since in this work we are only interested in the first two, in this section, we focus on summarizing the main existing methods adopted for their design.

Several models have been proposed in the literature to generate synthetic source signals for the auralization of passing vehicles. According to the ISO-362 standard, two separate components contribute to the sound produced by moving vehicles, namely the engine (or propulsion) noise and the road/tire interaction noise [23, 24]. These contributions are usually simulated separately using different techniques that can be categorized into physical modeling synthesis [11, 25–28], spectral-shaping [6, 17], recording-based synthesis [3, 13, 15, 29, 30], engineering methods [4, 5], and combinations of those [2, 12, 14, 31].

Physical modeling techniques have been used for the generation of both road/tire [25] and propulsion [11] noise. These methods consist in the definition of a parametric model of the physical behavior of the sound generation process. For road/tire interaction noise, the SPERoN and HYRoNE models have been proposed [25], based on deterministic calculations of the contact forces between the rubber surface of the tire and the asphalt road, eventually complemented with a statistical description of the non-linear dependency of the contact pressure on the road profile. Notwithstanding their elevate accuracy, these models require a difficult parameter tuning and come with a high complexity that hinders their practical usage for auralization. For propulsion noise, a physical model of the four-stroke engine has been developed, known as the Baldan model [11]. This relatively small model involves few parameters that describe the engine physics (e.g., number of cylinders, engine speed and load) and can be used to emulate the sound produced by different vehicle types (cars, trucks, motorbikes) with real-time capabilities.

Spectral-shaping synthesis techniques aim at designing the spectrum of the target signal using additive or subtractive synthesis, or a combination of both (spectral modeling synthesis). In [6], the propulsion noise component is generated using spectral modeling synthesis: this model employs a set of parameters to describe driving dynamics (engine speed, load and gear) and allows to simulate accelerating vehicles. As a main drawback, recordings performed in a controlled environment are needed to obtain the propulsion noise synthesizer parameters; this limits the applicability of the model to specific vehicle types for which high quality recordings are available.

Recording-based approaches rely on the analysis of one (or a set of) real recorded pass-by event(s) to generate a synthetic source signal to be used for simulation purpose. Two main techniques have been adopted. The first one consists in extracting the source signal by inverse-filtering a recorded pass-by event [15] to remove the effects of propagation, air absorption, ground reflection, source directivity, and Doppler shift and thus obtain a stationary signal that contains both contributions from road/tire interaction and propulsion. This method depends on the availability of a pass-by recording from which the inverse filters parameters can be extracted, and the generated signal is tailored to match the recording conditions. To extend the design space, granular synthesis has been proposed [29]. This method relied on the analysis of a large set of recordings to extract the grains needed to synthesize different vehicles, roads, speeds, and gears. The lack of large recording datasets constitutes the main drawback of these techniques.

Engineering models, such as Nord-2000 [4] and Harmonoise [5], aim at estimating the average sound pressure levels produced by passing vehicles. These methods describe both road/tire and engine noise using empirical equations, whose parameters have been extracted by regression analysis performed on a large dataset of traffic sounds, and are available in tables. The two source signal components are synthesized by filtering broadband noise using one-third octave filterbanks to match the sound pressure levels predicted by the aforementioned equations. These simple models are recording-free and produce accurate results for the simulation of the road/tire interaction sound. However, the averaging operation causes the engine sound to lack in texture (rattling, rumbling and tonal components) compared to other methods.

Finally, hybrid approaches have also been proposed. In [31], the Harmonoise model is used to simulate the road/tire interaction noise, and a modified version of the Baldan model [11] is adopted for engine simulation: this combination leads to an improved accuracy as compared to the standard Harmonoise technique, without significantly impacting the computational load. In [14], instead, the inverse filtering method [15] is combined with the Baldan [11] model with the same purpose.

For the simulation of acoustic propagation, existing simulators targeting traffic auralization rely either on frequency-domain processing [5, 17], time-domain processing [6, 16, 31], or a combination of both [9, 29].

Frequency-domain techniques, introduced in [5, 17], are designed with the aim of simulating propagation effects including geometrical spreading, air absorption, attenuated ground reflections, and source directivity by analyzing their individual impact on the sound pressure levels produced by the source in one-third octave frequency bands. The effects are applied as sound pressure level correction factors, depending on the relative position of the source and the receiver, at the source signal generation time. Sound synthesis is performed on short-time windows in which the source is assumed to be static.

Different techniques use a combination of time- and frequency-domain processing to consider the Doppler effect [9, 29]. In [29], the synthesized time-domain source signal is fed to a fractional delay line to model acoustic delay and Doppler effect. The delayed signal is then processed via frequency-domain filters to apply frequency-dependent gains to simulate sound attenuation and source directivity.

The last group of propagation methods contains techniques entirely based on time-domain processing of the signal generated by the source synthesis module [6, 16, 31]. These methods rely on the use of fractional delay lines to simulate acoustic propagation delay and Doppler effect for both the direct sound and the reflection from the ground surface, whereas the attenuation due to ground and air absorption are implemented as FIR filters in the time domain. These techniques come with a low computational complexity and acoustic propagation is decoupled from the source signal generation.

In our previous work [16], we introduced the open-source *pyroadacoustics* simulator, that implements sound propagation on a road by means of variable length delay lines and FIR filtering. The model, described in Section 3.2, allows to simulate the direct sound emitted by an omnidirectional source and the reflection from the ground surface, together with sound attenuation due to spherical propagation and air absorption, and comes with a low computational complexity.

In this paper, we extend pyroadacoustics to build an end-to-end framework for the simulation of vehicle pass-by events. *TrafficSoundSim* is implemented as an open-source python package [32] and can be used to simulate the sound produced by both cars and heavy vehicles (HV). This work introduces the following contributions:

1. We propose a *modular* structure that combines state-of-the-art source signal generation methods, namely the Harmonoise [5] road/tire interaction noise model and the Baldan physical engine noise model [11], with a sound propagation module built on the pyroadacoustics simulator. The modularity guarantees that all propagation effects (sound attenuation, air absorption, asphalt reflection, moving source directivity) are independent on the source signal generation, leaving the proposed framework open for extension to account for different source types (e.g., electrical engines).
2. We introduce an improved ground reflection model in pyroadacoustics, based on the *Delany and Bazley* model [22], and show that it results in an improved realism of synthesized sounds in terms of both error and psychoacoustics metrics.
3. We propose an implementation of the Harmonoise moving sound source directivity based on digital filtering performed in the propagation module. This implementation respects the modular structure of the proposed method and enhances extensibility to different sound sources.
4. We introduce in pyroadacoustics an implementation of common directivity patterns for sound sources and receivers.

To provide an overview of the existing traffic sound simulation tools, in Table 1, we compare the features and capabilities of some of the discussed state-of-the-art methods, including *TrafficSoundSim*. The strength of the proposed framework lies in the modular, user-controllable and extensible propagation module, designed in a physically accurate way and allowing to generate signals for arbitrary receiver configurations (i.e., arbitrary multi-channel microphone array geometry and directivity). This feature, together with its open-source release, enables *TrafficSoundSim* to be used in several applications ranging from auralization to data generation for training automatic traffic monitoring systems [20]. To validate the model, a comparison between simulated and recorded pass-by events in semi-controlled conditions is shown in Section 4, and audio demos are provided as additional files. The comparison has been carried out using traditional error metrics and psychoacoustics sound quality measures, both expressing a close similarity between simulated and recorded signals. Moreover, *TrafficSoundSim* has been successfully used to generate data for training a neural network to target acoustic vehicle counting in [20]. Although this application is relevant to assess the effectiveness of *TrafficSoundSim*, its discussion is outside the scope of this work and we refer to [20] for the details.

**Table 1:** Comparison of features between state-of-the-art simulators and the proposed framework. Features include the adopted source model, how rolling and propulsion sounds are simulated, the type of trajectory that the source can travel upon, the inclusion of Doppler effect, reflections and air absorption, whether recordings are needed to extract auralization parameters (e.g., for generating source signal), real-time capabilities, allowed receiver geometry configuration and source code availability

Feature	Kaczmarek [17]	Maillard et al. [29]	Pieren et al. [6]	Fu et al. [31]	Dreier et al. [14]	*TrafficSoundSim*
Source model	Harmonoise	Rolling+propulsion	Modified Harmonoise	Harmonoise	Rolling+propulsion	Harmonoise
Rolling sound	Additive synth.	Granular synth.	Spec. modeling	Harmonoise	Inv. filtering	Harmonoise
Propulsion sound	NA	Granular synth.	Spec. modeling	Baldan	Baldan	Baldan
Trajectory	Rectilinear	Arbitrary	Arbitrary	Arbitrary	Arbitrary	Arbitrary
Doppler	Yes	Yes	Yes	Yes	Yes	Yes
Reflections	Ground	None	Ground	Ground, buildings	Ground, buildings	Ground
Air absorption	Yes	Yes	Yes	Yes	Yes	Yes
Need recordings	No	Yes	Yes	No	Yes	No
Real-time	Yes	No	No	Yes	Yes	No
Receiver	Binaural	Mono, stereo, ambisonics, binaural	Multi-channel	Binaural	Arbitrary	Arbitrary
Open-source	No	No	No	No	No	Yes

## Framework description

In this section, we describe the *TrafficSoundSim* framework by first introducing the source synthesis and propagation modules and then discussing the vehicle pass-by simulation set-up.

### Source signal synthesis

According to the Harmonoise model [5], two main components contribute to the sound produced by a passing vehicle, namely the road/tire interaction noise, caused by the rolling motion of the tires on the rough ground surface, and the propulsion noise, produced by the engine. Following this description, in the source synthesis module of *TrafficSoundSim*, we separately generate the two components and combine them to create the source signals to be used as input for the propagation module. Note that the propulsion noise is mostly relevant for cars powered by thermal engines, whereas electric cars are not considered in our discussion. Nevertheless, since the generation of engine and rolling noise is decoupled, this structure would facilitate extending the proposed framework to electric vehicles as well.

#### Road/tire interaction noise

To synthesize the signal produced by road/tire interaction, we rely on the Harmonoise model [5]. Accordingly, the sound produced by the rolling tires has broadband characteristics (thus tonal components caused by resonances, as well as vibrations produced, e.g., by cobblestone pavements or non-smooth ground surfaces are not taken into account). The rolling noise component is generated by filtering white noise using a one-third octave filterbank with $N_b = 27$ bands, with center frequencies from 25 Hz to 10 kHz. The Harmonoise model predicts the SPL emitted in each band as a function of vehicle speed and type (i.e., light vehicles, medium vehicles, heavy vehicles and other vehicles), by means of the empirical equation

1 $$\begin{aligned} L_\text {WR}(v, f) = a_\text {R}(f) + b_\text {R}(f) \log (\frac{v}{v_\text {ref}})\,, \end{aligned}$$

where *f* is the frequency, $v$ is the speed of the vehicle, $v_\text {ref} = {70}\,\text {km/h}$ is a reference speed, and the regression parameters $a_\text {R}$ and $b_\text {R}$ are provided in one-third octave bands in the above mentioned frequency range. Correction coefficients, although not incorporated in the current description for the sake of clarity in presentation, can be introduced to accommodate variations in road surfaces and atmospheric conditions [2]. Moreover, a correction factor depending on the instantaneous source position can be used to model source directivity: however, to keep the source signal generation decoupled from the propagation effects, directivity is implemented in the propagation module in *TrafficSoundSim*. The discrete time-domain road/tire interaction noise signal is then computed as

2 $$\begin{aligned} s_\text {R}[n] = \sum \limits _{i=1}^{N_b} p_0 10^{\frac{L_\text {WR}(v,f_i)}{20}}\cdot s_{\text {noise},i}[n]\,, \end{aligned}$$

where $p_0 = 2\times 10^{-5}\,\text {Pa}$ is the hearing threshold and $s_{\text {noise},i} [n]$ is the white noise in the frequency band *i*. In practice, the signal $s_{\text {noise},i}[n]$ is obtained by passing a broadband white noise signal $s_\text {noise}[n]$ through the above-mentioned one-third octave filterbank. We underline that, differently from [31], we do not apply road surface correction factors, nor directivity correction factors. The latter, in particular, will be implemented in the propagation stage using digital filters, as discussed in Section 3.2.2.

#### Propulsion noise

For the synthesis of propulsion noise, we adopt the Baldan model [11], a physical model of the 4-stroke engine that allows to generate a realistic-sounding engine with a low computational complexity. This method is based on modeling three separate components that are responsible for most of the emitted sound:

1. The intake system is responsible for letting a mixture of air and fuel into the cylinder chamber via a valve that opens at regular time intervals. Acoustically, this is simulated by means of a digital waveguide [33] with fixed feedback on the free end and variable feedback on the valve end, connected to the cylinder. The intake system receives its primary signal from the cylinder component and is fed at the free end with a secondary signal consisting of a low-pass filtered white noise, that represents the turbulence in the air/fuel mixture caused by the aspiration from the opening valve. The free end yields the first output of the model, represented by the signal $s_\text {in}[n]$.
2. One or more cylinders with periodic motion, together with the fuel ignition mechanism, constitute the core of the engine. These components are controlled by the two main input parameters of the model, namely the engine speed (expressed in revolutions per minute―RPM) and by the engine load, and produce the tonal components of the engine, together with the rattling sounds produced by mechanical vibrations. The cylinders are connected to a second model output that represents the engine vibrations, represented by the signal $s_\text {vibr} [n]$.
3. The exhaust system represents the component that expels gas into the environment. It is simulated by a set of digital waveguides that model the exhaust pipes, the muffler elements, and the final outlet, fed with filtered white noise to represent the gas flow, and is connected to the cylinder module. The exhaust yields the third output of the model, represented by the signal $s_\text {ex} [n]$. Note that for the simulation of a passing vehicle, the exhaust system constitutes the major contribution to the overall produced engine noise [2].

Finally, the engine sound is obtained by summing the three components as

3 $$\begin{aligned} s_\text {E} [n] = A_1 s_\text {in}[n] + A_2 s_\text {vibr} [n] + A_3 s_\text {ex}[n]\,, \end{aligned}$$

where $A_1, A_2$ and $A_3$ represent relative amplitude weights. As the Baldan model does not provide guidelines to tune these parameters, they can be tuned manually according to the desired listening position [2].

#### Source signal generation

To generate the source signals that will be provided to the propagation module, we combine the road/tire and engine noise components following Harmonoise [2]. Accordingly, passing vehicles can be modeled using two vertically stacked point sources that emit a mixture of road/tire and propulsion noise. The lower source (LS) is located at a height of ${0.01}\,\text {m}$ for all types of vehicles, whereas the higher source (HS) is located at 0.3 m for cars and 0.75 m for heavy vehicles such as trucks and buses. The road/tire and propulsion signal powers are split with relative weights of [0.8, 0.2] between the two sources, with the LS containing most of road/tire noise power and vice versa, as [2]

4 $$\begin{aligned} s_\text {LS} [n] & = \frac{1}{\sqrt{5}} \tilde{s}_\text {E} [n] + \frac{2}{\sqrt{5}} s_\text {R} [n]\nonumber \\ s_\text {HS} [n] & = \frac{2}{\sqrt{5}} \tilde{s}_\text {E} [n] + \frac{1}{\sqrt{5}} s_\text {R} [n] \,, \end{aligned}$$

where $s_\text {LS}[n]$ and $s_\text {HS}[n]$ represent the time-domain signal assigned to the lower and higher source, respectively, $s_\text {R} [n]$ represents the road/tire noise signal generated as in (2) and $\tilde{s}_\text {E} [n]$ is a modified version of $s_\text {E} [n]$ defined in (3), normalized to the Harmonoise predicted noise power. To combine the signals as in (4), in fact, the relative power of the two components generated from road/tire interaction and propulsion must match the power predicted by Harmonoise.

We normalize the engine noise signal to match the Harmonoise engine power following the procedure described in [2]. First, we compute the engine noise predicted by Harmonoise: similarly to (1), the SPL produced by the engine noise can be computed as

5 $$\begin{aligned} L_\text {WP} (v, f) = a_\text {P} (f) + b_\text {P} (f)\log {\frac{v-v_\text {ref}}{v_\text {ref}}}\,, \end{aligned}$$

where the regression coefficients $a_\text {P}$ and $b_\text {P}$ are given in one-third octave bands. We then compute the time-domain Harmonoise engine signal as

6 $$\begin{aligned} s_\text {P}[n] = \sum \limits _{i = 1}^{N_b} p_0 10^{\frac{L_\text {WP}(v,f_i)}{20}}\cdot s_{\text {noise},i}[n]\,, \end{aligned}$$

where $s_{\text {noise},i}[n]$ is a subband white noise signal in the frequency band *i*, obtained by passing $s_\text {noise}[n]$ through a one-third octave filterbank with $N_b = 27$ bands, similarly to (2). Although the signal $s_\text {P} [n]$ can be directly used as engine noise source signal, it lacks the texture of a real engine: being a sum of filtered white noise components, it does not contain tonal components nor rattling and rumbling sounds produced by mechanical vibrations [2]. In *TrafficSoundSim*, instead, we exploit $s_\text {P}[n]$ to calibrate the power of the more accurate $s_\text {E}[n]$ to match the Harmonoise predictions and be able to apply (4). Therefore, we compute the normalized engine noise $\tilde{s}_\text {E}[n]$ as

7 $$\begin{aligned} \tilde{s}_\text {E} [n] = s_\text {E} [n] \cdot \frac{\sum \nolimits _{i = 0}^{N_s} \vert s_\text {P}[i] \vert ^2}{\sum \nolimits _{i = 0}^{N_s} \vert s_\text {E}[i] \vert ^2} \end{aligned}$$

where $N_s$ is the total length of the signals $s_\text {P}$ and $s_\text {E}$. The signals obtained using (4) represent the output of the source synthesis module, and are passed over to the propagation module.

### Sound propagation

**Fig. 1:**
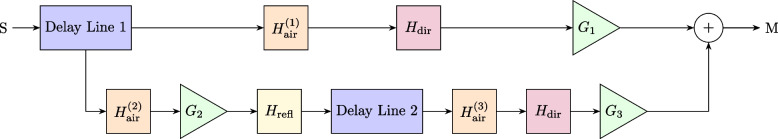
The architecture of the proposed propagation module: acoustic delay and Doppler effect are simulated using delay lines; air absorption for the paths source-receiver, source-reflection point on ground surface and reflection point-receiver, as well as ground reflection effect are simulated via FIR filters ($H_{\text {air}}$ and $H_{\text {refl}}$, respectively); $H_{\text {dir}}$ models sound source directivity in accordance with the Harmonoise description; attenuation caused by spherical propagation is modeled via gain elements $G_1, G_2, G_3$

The proposed sound propagation module, whose architecture is depicted in Fig. 1, is built on top of our open-source *pyroadacoustics* simulator [16], available at [34]. *Pyroadacoustics* is designed to emulate the propagation of the sound emitted by a single source moving with arbitrary speed and trajectory on a non-urban road, in which the main reflecting surface is represented by the ground. An arbitrary number of stationary virtual microphones, arbitrarily distributed in the space, provide the output of the simulator (i.e., they constitute the listening positions).

**Fig. 2:**
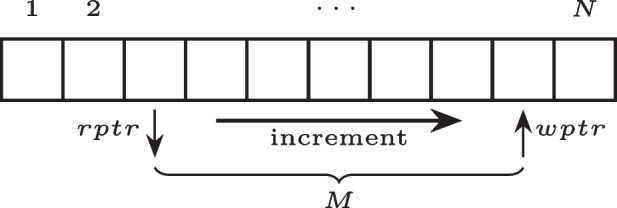
Circular buffer implementation of an *M*-sample delay line, with read (*rptr*) and write (*wptr*) pointers moving in the direction specified by the arrow [16]

**Fig. 3:**
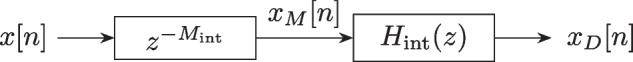
Implementation of delay line with fractional delay $M = M_{\text {int}} + \Delta$, where $M_{\text {int}} = \lfloor M \rfloor$ is the integer part and $\Delta \in [0,1)$ is the fractional part: the integer delay line of $M_{\text {int}}$ samples is followed by an interpolator $H_{\text {int}}$ [16]

In *pyroadacoustics*, the propagation delays, together with the Doppler effect, are implemented by means of variable length delay lines. Delay lines are simple signal processing blocks used to introduce a delay on the input signal they are provided with and can be implemented using a circular buffer with a defined length *N*, as depicted in Fig. 2. The input signal is written in the buffer at the position of a write-pointer (*wptr*) that gets incremented by one every computation instant; the output is read at the position of a read-pointer, delayed by *M* samples with respect to the write-pointer. The delay *M* can vary over time as long as the condition $M \le N$ is met, yielding a *variable length* delay line, that can be used to simulate acoustic propagation when relative motion is present between a sound source and a receiver. Given a signal *x*[*n*] emitted by a moving source, whose instantaneous position is denoted by $\varvec{r'}[n]$, and the signal *y*[*n*] received by a static listener at position $\varvec{r}$, the corresponding delay *M*[*n*] is given by

8 $$\begin{aligned} M[n] = \frac{\left\| {\varvec{r} - \varvec{r'}[n]}\right\| _2}{c}f_s\,, \end{aligned}$$

where $f_s$ denotes the sampling frequency, *c* is the speed of sound in the air, and $\left\| {\cdot }\right\| _2$ is the vector $\ell _2$-norm. For the clarity of the presentation, the time index *n* will be omitted from the expression of the delay *M*[*n*] that will anyhow always be considered time-dependent. Since *M* can take non-integer values in a real scenario (e.g., when the relative distance between a moving source and a receiver varies continuously), interpolated reads can be performed using an interpolation filter cascaded to the delay element, as depicted in Fig. 3. Linear, all-pass and sinc interpolation filters have been implemented in *pyroadacoustics*. Note that, since the position of the read and the write-pointer is tied to a physical model of sound propagation, a minimum (and maximum) delay interval is always present between them; thus, the read-pointer never surpasses the write-pointer [33]. The variable delay, together with the interpolated reads, guarantees that the Doppler effect is simulated without any further processing, making this architecture effective for the accurate simulation of fast moving sources. In *pyroadacoustics*, two delay lines are employed: the first one has two read-pointers and is used to model the direct path and the path connecting the source position to the reflection point on the ground surface. The second one, instead, is used to model the path from the reflection point to the microphone position.

**Fig. 4:**
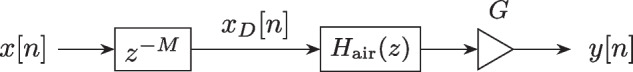
Simulation scheme of acoustic propagation: the input signal *x*[*n*] is passed through a variable length delay line, an attenuation filter to emulate air absorption, and a gain element to account for spherical propagation [16]

The delayed and interpolated signal $x_D[n]$ that exits a delay line is passed through a FIR filter $H_\text {air}$ that emulates the distance- and frequency-dependent effect of air absorption, and an attenuation gain *G* is added to account for spherical propagation (see Fig. 4). The air absorption filter is designed from its amplitude response, derived from the air absorption model described in the ISO 9613-1 standard [35], as discussed in [16].

#### Ground effect

The sound reflection produced by the ground surface is modeled in the propagation module, as shown in Fig. 1. In particular, sound reflection is implemented as a time-variant FIR filter $H_{\text {refl}}$ that takes as input the sound emitted by the source, delayed and filtered according to the distance between the source and the reflection point, computed using the law of reflection. In [16], the filter design originates from the real-valued, normal-incidence reflection coefficient: although the method is lightweight and allows to characterize several ground surface materials for which normal-incidence reflection coefficients are available [36], it neglects the phase effects caused by the complex impedance of rigid surfaces. To overcome this limitation, we hereby introduce an improved model of ground reflection, inspired by [8] and based on the *Delany and Bazley* model [22, 37].

To model the ground effect we assume a single, specular reflection path (i.e., scattering and diffusion are not included) and a locally reacting ground surface, implying that the complex-valued ground impedance *Z* does not depend on the angle of incidence $\vartheta$. The plane wave reflection coefficient is defined as [38]

9 $$\begin{aligned} R(\vartheta ) = \frac{Z \cos {\vartheta } - Z_0}{Z \cos {\vartheta } + Z_0}\,, \end{aligned}$$

where $Z_0$ is the characteristic impedance of air and *Z* is the complex-valued impedance of the road surface that can be expressed using the single-parameter Delany Bazley model as [22]

10 $$\begin{aligned} Z = 1 + 9.08\left( \frac{f}{\sigma }\right) ^{-0.75} - 11.9\jmath \left( \frac{f}{\sigma } \right) ^{-0.73}\,, \end{aligned}$$

where $\jmath$ denotes the imaginary unit, while the parameter $\sigma$ indicates the flow-resistance and can be computed for different materials. The spherical wave reflection factor can then be expressed as [37]

11 $$\begin{aligned} Q(\vartheta ) = R(\vartheta )\left( 1- R(\vartheta )\right) F(w)\,, \end{aligned}$$

where *w* represents the numerical distance

12 $$\begin{aligned} w = \sqrt{\jmath kd\left( 1 + \frac{1}{Z}\cos {\vartheta } - \sqrt{1 - \left( \frac{1}{Z}\right) ^2}\sin {\vartheta }\right) } \,, \end{aligned}$$

*d* being the distance between the source and the reflection point and $k = 2\pi f / c$ being the wave number. Finally, the function *F*(*w*) of the numerical distance *w* is defined as

13 $$\begin{aligned} F(w) = 1 + \jmath \sqrt{\pi }we^{-w^2}\textrm{erfc} (-\jmath w)\,, \end{aligned}$$

where we used the complementary error function

14 $$\begin{aligned} \textrm{erfc}(\xi ) = 1 - \frac{2}{\sqrt{\pi }}\int _{0}^{\xi } e^{-\chi ^2}\,d\chi \,. \end{aligned}$$

This method allows us to compute the complex-valued reflection coefficient $Q(\vartheta )$ for frequencies in the range $[0, f_s /2]$. We then implement the ground reflection effect using a FIR filter, by interpreting $Q(\vartheta )$ as the frequency response of the desired filter, and using the inverse Fourier transform to obtain its impulse response.

#### Directivity model

**Fig. 5:**
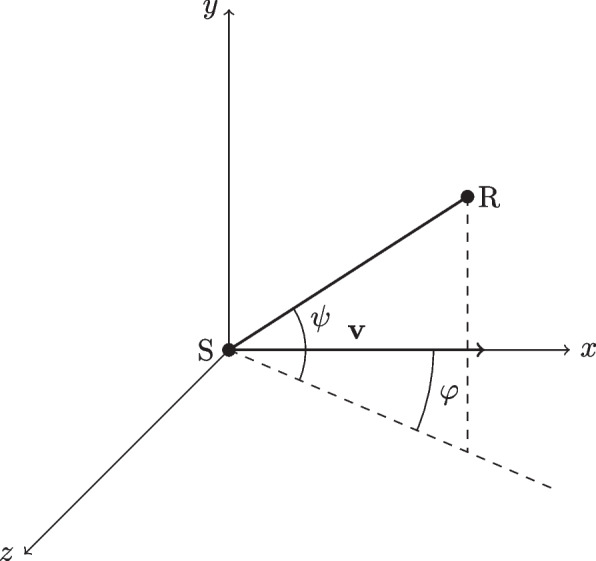
Geometry for the derivation of the directivity functions: $\textrm{S}$ denotes the sound source, moving at speed $\varvec{v}$, while $\textrm{R}$ denotes the static receiver

The Harmonoise method includes a model of horizontal source directivity, representing the *horn* effect produced by the rolling wheels, and vertical source directivity, to account for the screening effect caused by the car body [5]. Both the directivity effects are defined as SPL correction factors applied during the source signal generation, governed by closed-form functions of the horizontal angle $\varphi$ and vertical angle $\psi$ depicted in Fig. 5. Since the angles $\varphi , \psi$ depend on the relative position of the car and receiver that varies over time, the directivity computation is performed in each synthesis-window and is thus entangled to the signal generation.

To keep the source synthesis and propagation modules uncoupled, we propose an implementation of horizontal directivity based on FIR filtering in the propagation module (see Fig. 1). Since we consider a scenario where the receiver is located at the side of a road, at a relatively small height (smaller than 3 m), the vertical directivity has a minor contribution on the overall signal and, hence, it is currently not included in *TrafficSoundSim*. Note that, to consider sensors located on top of the road or listeners at higher positions (e.g., on high buildings or aircrafts), the impact of vertical directivity would instead be non-negligible and, thus, should be implemented. The flexibility of the framework and its open-source distribution easily allow this extension.

According to Harmonoise, the horizontal directivity always affects the signal emitted by the LS, while it is relevant for the HS only for heavy vehicles [5]. For the LS, the directivity correction coefficient, expressed as a variation in the SPL in one-third octave frequency bands, is defined as

15 $$\begin{aligned} \Delta L_H \left( \varphi , \psi \right) = \left\{ \begin{array}{l} \left( -2 .5 + 4|{\cos {\varphi }}|\right) \sqrt{\cos {\psi }} \quad f \in B \\ 0 \qquad \text {otherwise}\,, \end{array}\right. \end{aligned}$$

where $B = [800, 6300]$ Hz. We implement this effect by using a time-varying bandpass filter added to the propagation module. In particular, for each simulation instant:

1. The angles $\varphi , \psi$ are computed based on the (instantaneous) relative position of the source and receiver;
2. A bandpass FIR filter $H_{\text {dir}}(\varphi , \psi )$ is designed, with gain in the pass band computed using (15). Note that, to reduce the computational burden of computing a filter for each simulation instant, directivity filters are pre-computed and stored in a table when initializing the framework;
3. The source signal is filtered using $H_{\text {dir}}(\varphi , \psi )$.

For the highest source in HVs, the directivity correction is expressed as [5]

16 $$\begin{aligned} \Delta L_H \left( \varphi , \psi \right) = &(1.546(\pi /2 - \varphi )^3 - 1.425(\pi /2 - \varphi )^{2} \\ & + 0.22(\pi /2 - \varphi ) + 0.6 )\sqrt{\cos {\psi }}\,, \end{aligned}$$

that can be implemented as a gain element in the propagation module (since it does not depend on frequency but only on the angles $\varphi , \psi$).

In addition to the source directivities, first-order common microphone directivity patterns have been implemented. Assuming the receiver directivity to be invariant to rotations with respect to the horizontal plane, the first-order directivity pattern is described by the formula

17 $$\begin{aligned} D(\alpha ) = a + (1 - a) \cos {\alpha }\,, \end{aligned}$$

where $\alpha$ represents the angle between the orientation of the microphone and the line connecting it to the position of the source, and *a* is a parameter that can be used to define different patterns (e.g., omnidirectional, $a = 1$, subcardioid, $a=0.75$, cardioid, $a=0.5$, supercardioid $a=1/3$, hypercardioid, $a=0.25$, and figure-8, $a=0$).

### Simulation setup

**Fig. 6:**
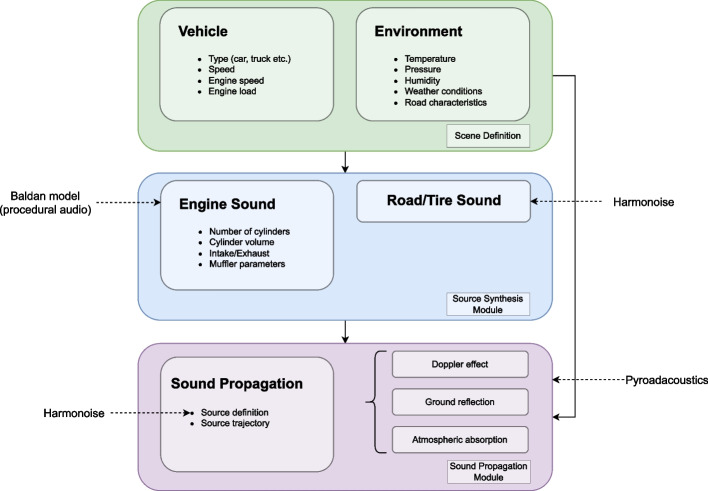
*TrafficSoundSim* is composed of three blocks: the interface of the model that collects the description of the acoustic scene to be simulated; the source synthesis module, responsible for the generation of source signals based on the provided input parameters; the sound propagation module, simulating acoustic propagation and producing the output signal

The overall structure of *TrafficSoundSim* is depicted in Fig. 6. The simulator consists of three independent blocks that communicate via shared parameters and features a unidirectional signal flow.

The first block constitutes the interface of *TrafficSoundSim* and is responsible for the collection and storage of the parameters to set up the simulation. These parameters include (i) environmental parameters (temperature, pressure, relative humidity, flow resistivity of the ground surface material), (ii) microphone parameters (position and directivity pattern of each microphone), and (iii) source parameters (type of vehicle―car or HV―trajectory, speed, engine speed, engine load). The parameters are shared among the blocks.

The second block constitutes the source synthesis module, responsible for the generation of source signals, as described in Section 3.1. The road/tire interaction noise generation model has been implemented in Python as part of *TrafficSoundSim*, whereas for the Baldan model, adopted to generate engine noise, we rely on an open-source Rust implementation [39]. The road/tire interaction and engine noise are then combined according to the Harmonoise model to produce the source signals of the LS and HS that are then handed over to the third block.

The third and last block constitutes the propagation module, responsible for the simulation of acoustic propagation, air absorption, ground effect, and source directivity, as described in Section 3.2. This module takes as input the LS and HS signals and propagates them in the scene defined in the first block: variable-length delay lines are used to simulate Doppler effect and acoustic delay, whereas ground reflection, air absorption, and source directivity are implemented using FIR filters. This block returns a single-channel signal for each microphone present in the acoustic scene.

## Results and discussion

To illustrate the capabilities of *TrafficSoundSim*, we run simulations of pass-by events and compare synthetic and real signals. The simulations are designed starting from real-world labeled recordings, collected by Bosch on a two-lane German municipal road. The recordings have been obtained with an omnidirectional microphone deployed on the sidewalk, at 2.7 m height and 3.5 m horizontal distance from the closest edge of the closest lane. The recording microphone has a flat magnitude response in the frequency range $f \in [100, 5000]$ Hz. For higher frequencies, as a consequence of the microphone enclosure, a lowpass filtering effect and some artifacts caused by resonance are observed. The recorded audio is accompanied by metadata for each passing vehicle, including its type (car or HV), speed, pass-by timestamp, and direction of transit (vehicles drive from left to right in the closest lane, and from right to left in the other one). Using this information, we set up simulations that emulate the target recordings: to this end, we assume that vehicles have a constant speed throughout the pass-by, as no turns, crossroads, or traffic lights are present nearby the microphone position; moreover, we only consider recordings in which isolated events are registered. We define an event to be isolated if no other pass-by is registered in an interval of 60 s temporally centered on the pass-by instant (i.e., the moment in which the vehicle is closest to the microphone). A total of 5 isolated pass-by events have been identified for each class (car and HV) and used to run simulations for evaluation purposes.

To generate synthetic pass-bys, we use the metadata to set up simulations using the procedure described in Section 3.3. We adopt a sampling frequency $f_s = {16}$ kHz and set the following parameters for the definition of the acoustic scene: a temperature $T= {20}^{\circ }$C, in line with the average day temperature of Southern Germany in Autumn, when the data was collected; an asphalt flow resistance $\sigma = 20\,000\ \cdot 10^3\,\text {Ns/m}^{-4}$ [40]. We heuristically set the atmospheric pressure $p = {1}$ atm and the relative humidity to $50\%$. To assess the source synthesis module, we simulate each pass-by event using three different models. First, we define a *baseline* (BL) model that represents each vehicle using a single point source with a height corresponding to the Harmonoise LS (i.e., 0.01 m). To generate the BL source signal, we use linear predictive (LP) modeling [41] to match the BL spectrum with that of a 1-s-long segment of the recorded signal considered in each experiment, starting 1 s after the pass-by instant (in order to avoid modeling the prominent Doppler shift around the pass-by instant). To this end, we design a third order linear predictor by analyzing the segment of the recorded signal and use it to filter a white noise signal to create the spectrally matched source signal. We then define the *Harmonoise* (HM) model, corresponding to the characterization of each vehicle using a HS and a LS, as described in Section 3.1.3; in this model, the engine and tire/road components are generated by filtering white noise as described, respectively, in (2) and (6) [5]. Finally, the *Harmonoise + Baldan* (HM+BD) model denotes the complete source signal generation model described in Section 3.1. Here, the road/tire interaction noise is generated via the Harmonoise synthesis process (see Eq. (2)), while the propulsion noise is generated using the Baldan model.

Since no information on the engine characteristics is available for the recorded pass-bys, we choose to use 4 cylinders for cars, 6 for HVs, and we manually tune the engine speed and load parameter. Moreover, we set the amplitude parameters of the three engine components (intake, exhaust and vibration) introduced in (3) as follows: since the vibration component mostly contributes to the low-frequency rumbling noise perceived in the cabin of the vehicle, and we consider for the current simulations a recording position located outside of it, where its impact is limited, we set $A_2 = 0.1$ in all simulations. As done for the engine speed and load, we manually tune $A_1$ and $A_3$. After defining the trajectories (i.e., start and end point, direction of movement) using the labels, we simulate 50-s-long audio segments. To match the microphone magnitude response, we apply a second-order bandpass filter to all simulated signals, with bandpass range between 100 Hz and 5000 Hz.

To assess the effectiveness of *TrafficSoundSim*, we first analyze the power spectral density (PSD) computed using the Welch’s method [42]. Accordingly, given an audio signal *x*[*n*], we first compute the periodogram as [43]

18 $$\begin{aligned} P_x(m,\omega _k) & = \frac{1}{M_w} |{\textrm{FFT}_{N_{\text {fft}},k}(x[n])}|^{2} \\ & = \frac{1}{M_w} \left|\sum \limits _{n=0}^{N_{\text {fft}}-1} x[n]w[n+mR]e^{-\jmath \frac{2\pi nk}{N_{\text {fft}}}}\right|^2\,, \end{aligned}$$

where $\omega _k$ is the *k*th frequency bin, the *m*th frame of signal *x*[*n*] is extracted using the $M_w=2f_s$ sample-long Hann window *w*, $R=f_s/2$ denotes the hop size, and $\textrm{FFT}_{N_{\text {fft}}}$ is the $N_{\text {fft}}$-point fast Fourier transform (FFT), where we use $N_{\text {fft}} = M_w$. If we average $P_x$ over a defined number *K* of frames of *x*[*n*], we obtain the PSD

19 $$\begin{aligned} \textrm{PSD} (x, \omega _k) = \frac{1}{K} \sum \limits _{m=0}^{K-1} P_x(m, \omega _k)\,. \end{aligned}$$

We perform the evaluation as follows. First, we compute the $\textrm{PSD}$ of a 4-s-long audio segment extracted from the real and synthetic audio signals, temporally centered on the pass-by instant. This measure is used to evaluate the quality of the signal in a time interval close to the pass-by instant, thus excluding the signal tails (note that here we consider as tails the parts of the signal that are more than 2 s distant from the pass-by instant). Then, we trace a temporal stack of periodograms for the whole 50-s-long signal including tails, as discussed in the following. To quantitatively assess how close the simulated signals are to the recorded ones, we adopt two error metrics, namely the root mean squared error (RMSE) between the PSDs of two signals, defined in dB scale as

20 $$\begin{aligned} \textrm{RMSE}(x_{\text {sim}}, x_{\text {real}}) = & \left(\frac{1}{P} \sum \limits _{k=1}^{P} \left[ 20 \log _{10}(\textrm{PSD}(x_{\text {real}},\omega _k)) \right.\right. \\ & \left.\left. - 20 \log _{10}(\textrm{PSD}(x_{\text {sim}},\omega _k))\right] ^2 \right)^\frac{1}{2}\,, \end{aligned}$$

where *P* denotes the length of the PSD vector (i.e., the total number of frequencies on which the PSD is computed) and the mean absolute error (MAE) between the power spectral densities in dB

21 $$\begin{aligned} \textrm{MAE}(x_{\text {sim}}, x_{\text {real}}) = & \frac{1}{P} \sum \limits _{k=1}^{P} |20\log _{10}(\textrm{PSD}(x_{\text {real}},\omega _k)) \\ & - 20\log _{10}(\textrm{PSD}(x_{\text {sim}}, \omega _k)) |\,, \end{aligned}$$

Since most of the information is contained in the time interval around the pass-by instant, both metrics are computed on the 4 s-long signal in the following experiments.

To evaluate the perceptual quality of the simulated sounds, we also consider two psychoacoustics sound quality measures from the Zwicker’s method [44], namely the *roughness* and the *sharpness*. These sound quality measures are commonly used to evaluate drone noise annoyance [45] and traffic noise annoyance [46]. Roughness, measured in Asper, measures the subjective perception of rapid amplitude and frequency variation of sound; a high roughness is an indicator of an aggressive sounding acoustic signal [47]. Sharpness denotes the strength of high-frequency components in the sound and is measured in Acum; it can be interpreted as a measure of the color of the sound [48]. Both metrics are computed based on auditory models and correlate with human perception. Note that, since they are not error metrics, they are computed on individual audio signals, and the assessment is performed by analyzing the agreement between the sound quality measures obtained for the real and for the simulated signals. We used the implementation of metrics provided in [49].

### Experimental results

**Fig. 7:**
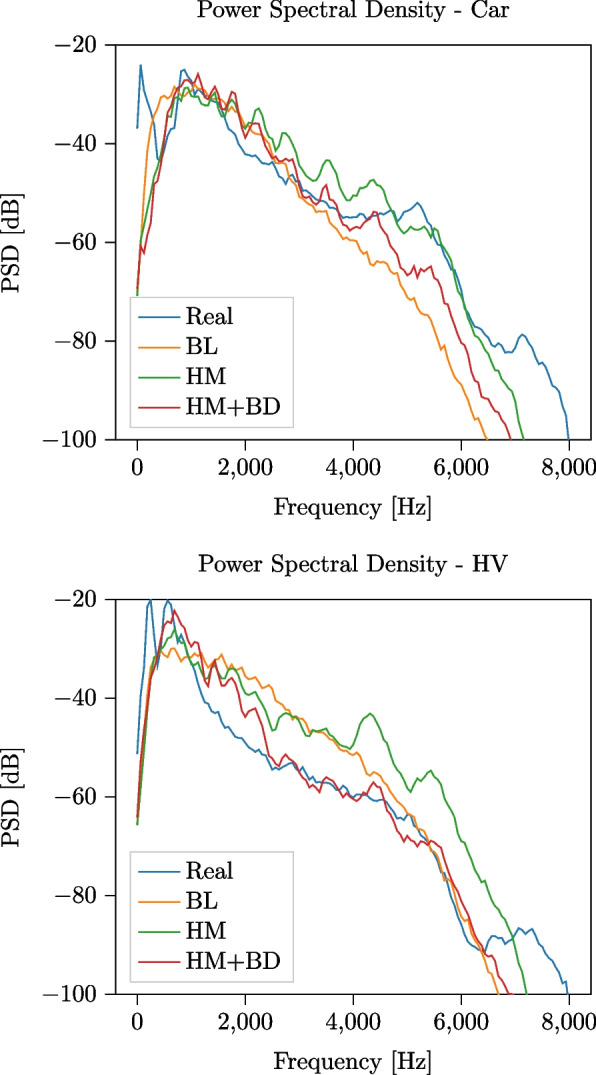
PSD computed on a 4-s signal temporally centered on the pass-by instant for the recorded event and the simulated events for a car (speed $v = {52}$ km/h) and HV ($v={56}$ km/h) using baseline, Harmonoise and combined Harmonoise + Baldan model for signal generation. The engine speed is set to $3000\,\text {RPM}$, and the propagation is the same for all simulations

We first qualitatively analyze the difference between the PSDs and the periodograms of a single pass-by event for each of the two classes. For the car vehicle, a speed of 52 km/h is used in accordance with the label of the recorded event, and for the Baldan engine model, a 4-cylinder engine is simulated, running at a speed of $3000\,\text {RPM}$. Similarly, for the HV a speed of 56 km/h and a 6-cylinder engine running at $3000\,\text {RPM}$ have been used. In Fig. 7, we show the PSD computed on a 4-s-long segment of the audio signals, temporally centered on the pass-by instant, comparing the real event and the three simulations (BL, HM, HM+BD). All models produce a PSD that closely matches the one of the recorded event in the frequency range $f \in [100, 5000]\,$Hz, with HM and HM+BD achieving the closest match and showing the capability of the models to accurately simulate a passing vehicle at constant speed. Note that, since the BL source signal is designed to match the spectral features of the recorded signal used for the experiment, a close match of the two PSDs is to be expected. At higher frequencies, some resonance artifacts caused by the microphone enclosure can be observed in the real event. However, their impact to the overall PSD is negligible, being their amplitude more than 30 dB lower than the amplitude registered at 5000 Hz. We also observe that, whereas for car events the HM and HM+BD show a comparable performance, the difference becomes larger when comparing HV events. In HV pass-bys, in fact, the engine component plays a more important role: therefore, the HM+BD simulation, including a more detailed engine texture, is better suited to simulate heavy vehicles.

**Fig. 8:**
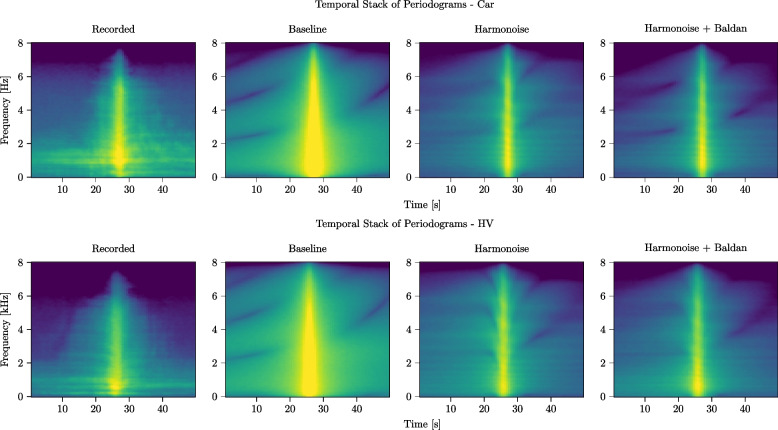
Temporal stacks of periodograms computed for passing car (speed $v = {52}$ km/h, engine speed $3000\,\text {RPM}$) and HV (speed $v= {56}$ km/h, engine speed $3000\,\text {RPM}$): comparison between recorded and simulated events using BL, HM, and HM+BD source signal generation models

In Fig. 8, we show temporal stacks of the periodograms of the full 50-s-long signals corresponding to the same events. The periodograms have been computed using a sliding window approach, on 2-s-long segments with an overlap of 500 ms. We observe that the recorded signals are spectrally richer than all of the simulations: this is due both to the presence of low-amplitude, broadband background noise that has not been included in the simulated signals and to the scattering and diffusion effects that are not modeled by the geometrical acoustic propagation model adopted in *TrafficSoundSim*. The difference is particularly evident in the signal tails, whereas around the pass-by instant, a better match can be observed. When compared to the BL, the HM and HM+BD models exhibit a closer match to the source signals, with tonal components (i.e., horizontal lines) corresponding to engine orders and resonances not appearing in the BL model. An artifact that can be observed in the simulations, particularly accentuated for the BL model, is the presence of deep valleys produced by the destructive interference caused by the ground reflection. This is attenuated in the recorded audio by the scattering effect, thus it is not visible in the real-world data. This illustrates the main limitation of the proposed propagation framework.

The results suggest that the proposed simulation framework, with either the HM or the HM+BD sound source signal, achieves a higher realism compared to the baseline, showing the importance of having an accurate sound source model for the simulation of traffic noise. Moreover, audio demos for these comparisons are available in the additional file set 1 (for the car events) and additional file set 2 (for the HV events). From these files, it is possible to evaluate the potential of the proposed framework and the impact of the choice of the sound source model. In the HM+BD model, the rumbling and rattling sound of the engine sound contribute to the realism of the simulation, at the cost of the manual tuning of the engine parameters.

To show how these results generalize to different pass-by events (i.e., recorded with different cars, having a different speed and different engine parameters), we then compare five 4-s-long pass-bys for each of the two classes and report the mean and standard deviations of the $\textrm{MAE}$ and $\textrm{RMSE}$ scores for the BL, HM, and HM+BD models in Table 2. The speed of the recorded events is in the range [52, 69] km/h, and, since no information is available on the engine parameters, we randomly selected an engine speed in the range $[2000,5000]\,\text {RPM}$. For the BL, a source signal spectrally matching the recorded one is generated for each considered event using LP filtering. Both metrics improve for all vehicle types when a more accurate source signal generation is used, showing the importance of adopting an accurate source model in the simulation of vehicle pass-by events. As an additional remark, in the generation of the propulsion signal, the engine parameters have been manually tuned and do not necessarily match the real conditions. Although a better tuning could lead to improved realism, these results suggest that even without an accurate knowledge (or tuning) of the engine parameters, difficult to fulfill in a real-world situation where this information is usually missing, a good match with recorded can be obtained. Finally, the benefit of using the physical engine model instead of the Harmonoise engine characterization is the improved realism of the source signal texture that includes rattling and rumbling effects, not captured by a broadband noise signal characterization.

**Table 2:** Mean and standard deviation of $\textrm{RMSE}$ and $\textrm{MAE}$ metrics computed for the BL, HM, and HM+BD models on 5 pass-by events for each vehicle type (car, HV). All metrics are expressed in dB scale

		BL	HM	HM + BD
Car	RMSE	$8.21 \pm 1.70$	$4.89\pm 1.20$	$\mathbf {4.05} \varvec{\pm } \mathbf {1.10}$
	MAE	$6.66 \pm 0.89$	$4.18\pm 1.06$	$\mathbf {3.38} \varvec{\pm } \mathbf {1.09}$
HV	RMSE	$8.30 \pm 1.12$	$6.77\pm 1.92$	$\mathbf {4.90} \varvec{\pm } \mathbf {1.03}$
	MAE	$7.10 \pm 0.78$	$6.05\pm 1.91$	$\mathbf {4.18} \varvec{\pm } \mathbf {0.96}$

To further assess the auditory plausibility of the proposed framework, the sharpness and roughness psychoacoustic metrics are computed for the 5 pass-by events in a 2-s time window centered on the pass-by instant and reported, for the recorded audio and the three simulations, in Table 3. This table shows that the BL achieves the closest match with the recorded audio, that might be explained by the fact that the source signal used for the BL is artificially shaped to closely match the recorded audio. In accordance to what observed when using error metrics, the table shows that HM+BD model comes closer to the recorded audio than the HM, suggesting that a more accurate engine model is beneficial to obtain a higher realism.

**Table 3:** Mean and standard deviation of roughness andsSharpness metrics computed for the real, BL, HM, and HM+BD models on 5 pass-by events for each vehicle type (car, HV)

		Real	BL	HM	HM + BD
Car	Roughness	$0.187 \pm 0.053$	$\mathbf {0.135 \pm 0.073}$	$0.083\pm 0.049$	$0.253\pm 0.012$
	Sharpness	$1.493 \pm 1.133$	$\mathbf {1.464 \pm 0.049}$	$1.646\pm 0.013$	$1.608\pm 0.061$
HV	Roughness	$0.165 \pm 0.042$	$\mathbf {1.530 \pm 0.028}$	$0.127\pm 0.024$	$0.135\pm 0.030$
	Sharpness	$1.459 \pm 0.054$	$\mathbf {1.433 \pm 0.040}$	$1.601\pm 0.013$	$1.562\pm 0.008$

The perceptual evaluation of the framework goes beyond the scope of this work; nevertheless, audio demos are available as additional material to show its capabilities. Even though simulations achieve a good match with recordings in terms of the shown metrics and sufficiently good auditory quality as from listening to the sounds, differences can be both heard in the audio files and seen in the PSD plots. The first major reason for that is the absence of any form of background noise in the simulation that constitutes a difference with real recordings. Secondly, the Harmonoise model adopted for the generation of rolling noise is not capable of simulating the so-called *horn effect* that impacts on the perceptual quality of the road/tire interaction sound. In order to account for that, more complex physical models of the interaction between the tire and the asphalt surface might be used, at the cost of significantly increasing the computational effort required by the simulator [25]. Lastly, in the propagation module, diffusion and scattering effects are not considered. These factors have an impact on the sound quality of the simulations, specially in the higher frequency range, and explain the auditory and spectral differences observed between the recorded and simulated audio.

**Fig. 9:**
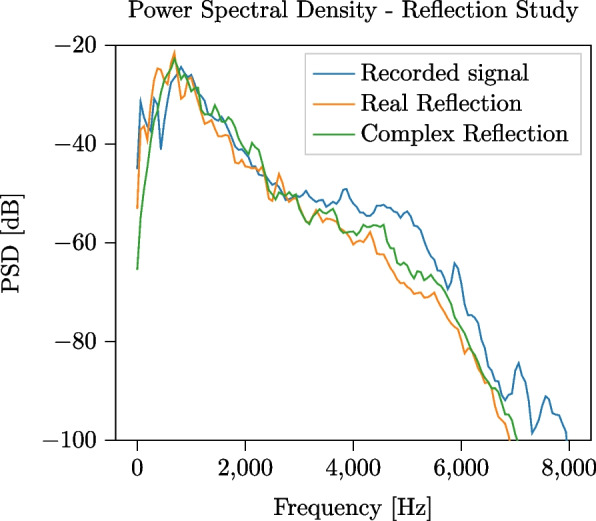
Comparison between PSD obtained using, respectively, real-valued and complex-valued reflection coefficients to model ground effect in the propagation module. Single HV pass-by event, with speed $v={61}$ km/h and engine speed $3000\,\textrm{RPM}$

In all the discussed simulations, a complex-valued reflection coefficient has been used to model ground reflection, as introduced in Section 3.2.1. To illustrate the benefit of using a complex-valued reflection coefficient, as compared to using a real-valued one, we report in Fig. 9 the PSD of two 4-s-long simulations generated using the HM+BD source signal model and the two different reflection models. Since the effect produced by the reflection is independent on the source signal model, we limit the analysis to the HM+BD model, without loss of generality. The metrics corresponding to these tests are reported in Table 4; the sound quality measures are reported in Table 5. From both the figure and the tables, it can be observed that modeling the ground reflection using a complex-valued reflection coefficient leads to a better match with the recorded pass-by, with a $\textrm{MAE}$ improvement of 1.78 dB and a $\textrm{RMSE}$ improvement of 1.35 dB. Moreover, the sound quality measures and the auditory evaluation of the audio demos, available in the additional file set 3, confirm this result. When a real-valued reflection coefficient is used, in fact, the effect of the ground reflection on the phase of the signal is neglected, whereas it is considered when the complex-valued reflection coefficient is used.

**Table 4:** Comparison between recorded pass-by and simulations run with real-valued and complex-valued ground reflection coefficient $R(\vartheta )$ for the same single HV pass-by event (speed $v={61}$ km/h, engine speed $3000\,\textrm{RPM}$) represented in Fig. 9. All metrics are expressed in dB scale

Metric	Real $\varvec{R(\vartheta )}$	Complex $\varvec{R(\vartheta )}$
RMSE	6.65	**4.87**
MAE	5.17	**3.82**

**Table 5:** Comparison of roughness and sharpness sound quality metrics computed for the real recorded signal and for simulations run with real-valued and complex-valued ground reflection coefficient $R(\theta )$. The considered event is a HV with speed $v={61}$ km/h, engine speed $3000\,\textrm{RPM}$

Metric	Recorded audio	Real $\varvec{R(\theta )}$	Complex $\varvec{R(\theta )}$
Roughness	0.173	0.381	**0.165**
Sharpness	1.524	1.389	**1.461**

We finally analyze the simulation time needed to create auralizations using the proposed method. In Table 6, we report the total time required by the proposed method to simulate 1 s, 10 s, 30 s, and 60 s of audio and break it down to the time needed to perform different parts of the simulation. The simulations run on a 8 core Apple M1 CPU, with $16\,\textrm{GB}$ memory. In the upper part of the table, we investigate the time needed to generate the source signal, to initialize the simulation, and to generate the direct and the reflected audio signals. Moreover, we report the additional time required by auxiliary functions, and not included in the listed components. In the lower part of the table, instead, we report the time needed to perform the air absorption, asphalt reflection and directivity filtering, when simulating both the direct and reflected path. Simulating only the direct path results in the fastest method, achieving real-time capability in the current implementation. Increasing the physical accuracy, by adding the reflected path, comes at the cost of a slower simulation. The current Python implementation of *TrafficSoundSim* might be optimized for simulation speed; this improvement is beyond the scope of this paper and is left for future work.

**Table 6:** Time in seconds required to simulate 1 s, 10 s, 30 s, and 60 s of audio, split into different simulation blocks. Upper part of the table: breakdown of total simulation time into source signal generation (HM+BD model), initialization, simulation of acoustic propagation along direct and reflected path and time spent into other auxiliary functions. The sum of these components adds up to the total time. Lower part of the table: time employed by absorption filtering, asphalt reflection filtering and directivity filtering throughout the entire simulation (i.e., direct and reflected paths)

Component	1 s audio	10 s audio	30 s audio	60 s audio
Source signal generation	1.092	1.290	1.733	2.654
Initialization	0.133	3.884	3.902	4.081
Direct path	0.567	5.597	17.010	33.998
Reflected path	2.292	22.044	64.795	127.276
Other (auxiliary)	4.311	5.719	17.370	34.765
Total time	8.395	38.534	104.810	202.774
Air absorption filtering	0.548	5.392	16.437	32.871
Asphalt reflection filtering	1.463	13.872	39.887	77.434
Directivity filtering	0.359	3.547	10.776	21.584

### Comparison with state of the art

In this section, we compare *TrafficSoundSim* with the state-of-the-art auralization framework [6], based on spectral modeling synthesis (SMS). This framework is specifically designed for the simulation of accelerating passenger cars and comes with an extensive set of adjustable parameters to maximize the realism of the auralizations in specific scenarios. Its internal structure consists of an emission module, gathering two submodules designed to generate road/tire noise and propulsion noise, a propagation filtering module and a final rendering module. Although the backbone of the emission module is based on the Harmonoise two-source model of a car vehicle, the source signals are generated using spectral modeling synthesis, and the parameters are obtained from an extensive set of recordings of several car models. The propagation module produces the effects of the propagation delay and spreading of the emitted sound, the Doppler effect, the convective amplification, the ground reflection, and air absorption; the implementation of these effects relies on delay lines and digital filtering. The key features of [6] are the possibility to realistically simulate accelerating vehicles and gear changes, as showcased in the published demos^1^^,^^2^. Its main drawbacks are that it requires recordings for the source signal generation, it is tailored to simulate passenger cars, hence not supporting different vehicles, and it is not publicly available.

Since [6] does not allow to generate truck simulations, we compare it with *TrafficSoundSim* based on the car pass-by event used for the experiments discussed in Figs. 7 and 8. To enable this comparison, the authors of [6] generated an auralization with their tool corresponding to the target scenario (described in the beginning of Section 4.1) and manually tuned the SMS simulator parameters based on the recorded audio considered in the comparison: the car drives in the 3rd gear, with an engine speed of 2700 RPMs, and the recording microphone is set at a height of 1.2 m. The SMS audio segment used in this evaluation is available in the additional files.

**Fig. 10:**
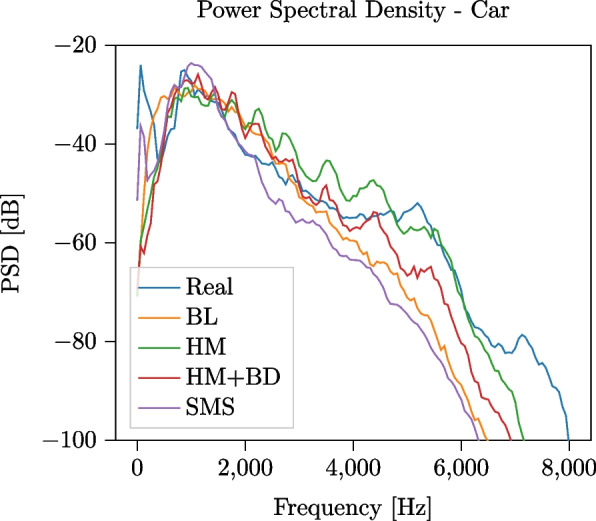
Comparison between PSD of 4-s-long recorded car pass-by event and simulations using baseline, *TrafficSoundSim* (Harmonoise and Harmonoise + Baldan configurations), and state-of-the-art simulator [6] based on modeling synthesis (SMS). The parameters of the SMS method are manually tuned based on the recorded signal: the car drives in 3rd gear with an engine speed of 2700 RPMs. The parameters of *TrafficSoundSim* are the same used in Fig. 7

**Fig. 11:**
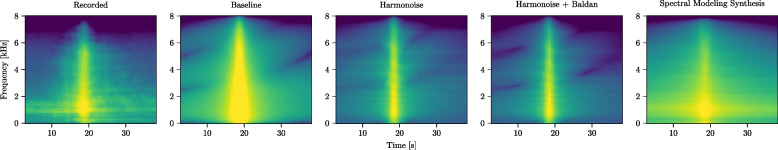
Comparison between temporal stacks of periodograms computed for a recorded car pass-by and simulations using baseline, *TrafficSoundSim* (HM and HM+BD) and SMS [6]. All parameters are the same used for Fig. 10

Following the experimental outline presented in Section 4.1, we first qualitatively compare in Fig. 10 the $\textrm{PSD}$s of 4-s-long segments of the recorded and simulated signals, centered around the pass-by instant. The SMS framework closely matches the recorded signal at frequencies lower than 2 kHz, whereas at higher frequencies the HM and HM+BD model achieve a closer match. In Fig. 11, we show temporal stacks of the periodograms of 40-s-long signals centered around the pass-by instant: this figure shows that the SMS framework captures the signal tails better than *TrafficSoundSim*, achieving a closer match with the recorded audio. Finally, we carry out a quantitative evaluation by computing error and psychoacoustics metrics on the 4-s-long segments centered on the pass-by instant, reported in Table 7. *TrafficSoundSim* achieves the best $\textrm{RMSE}$ score with the HM source model and the best $\textrm{MAE}$ score with the HM+BD source model, outperforming the SMS framework. In terms of psychoacoustics metrics, the best roughness score is obtained by the HM+BD model, whereas the best sharpness is obtained by the BL model.

**Table 7:** Comparison of error and psychoacoustics metrics for a recorded car pass-by event and simulations using baseline, HM, HM+BD, and SMS [6] simulators. All parameters are the same used for Fig. 10

Metric	Real	BL	HM	HM+BD	SMS
RMSE	-	8.65	**5.24**	5.28	10.99
MAE	-	6.48	4.60	**4.02**	8.47
Roughness	0.118	0.181	0.038	**0.160**	0.193
Sharpness	1.471	**1.401**	1.644	1.544	1.364

The analysis shows that *TrafficSoundSim* achieves competitive results when compared with the state-of-the-art. Although the audio demos and Fig. 11 show that [6] is more effective in modeling longer-term effects of the passing vehicle, the metrics reported in Table 7 and the short-term PSD plotted in Fig. 10 suggest that *TrafficSoundSim* achieves higher realism around the pass-by instant. Moreover, as underlined in Table 1, we remark that the two tools have different features and limitations that might make each of them the best option for some specific scenarios. Finally, the open-source distribution of *TrafficSoundSim* constitutes a major advantage over other tools not publicly available for usage.

## Conclusions

In this work, we have presented *TrafficSoundSim*, a framework for the acoustic simulation of passing vehicles that relies on the *pyroadacoustics* road acoustics propagation simulator. *TrafficSoundSim* contains two main modules, used for the generation of source signals and for the simulation of acoustic propagation. The source signal generation is based on the Harmonoise engineering method that characterizes a passing vehicle using two vertically stacked sources containing a mixture of road/tire interaction noise and engine noise. The road/tire interaction is simulated using broadband noise, filtered in one-third octave bands according to the Harmonoise description. The engine component, instead, is simulated using the Baldan model, based on physical modeling, to improve the sound quality over Harmonoise by including tonal components as well as rattling and rumbling sounds that characterize thermal engines. The propagation module is an extended version of *pyroadacoustics* that models sound propagation and Doppler effect using variable length delay lines and ground reflection and air absorption using FIR filters. In particular, we introduced an improved ground reflection model based on the use of a complex-valued reflection coefficient and implemented directivity for both sound sources, based on the Harmonoise directivity model, and for the virtual microphones, using standard directivity patterns. Lastly, we have shown that an accurate source signal generation can significantly improve the realism of the simulations by comparing simulated and recorded pass-by events for cars and heavy vehicles.

Some improvements of *TrafficSoundSim* can be foreseen for the future. First, a sound reproduction module based on head-related transfer functions can be implemented to build a full auralization framework. Second, although in the current version the only simulated reflection is the one produced by the ground surface, further reflections from buildings and objects on the road can be included by means of additional delay lines. Third, further study on source signal generation is required to characterize electric vehicles: the decoupled structure adopted for the generation of the rolling and propulsion noise in *TrafficSoundSim* facilitates its extension to include these vehicles by introducing a different model of the sound produced by their engine. Lastly, perceptual tests could be performed to further validate the framework.

## Supplementary Information

Supplementary Material 1.

## Data Availability

The source code is available at https://github.com/boschresearch/acoustic-traffic-simulation-counting. The following software that implements the Baldan model has been used in the work. Project name: enginesound v1.6; project home page: https://github.com/DasEtwas/enginesound; operating system: platform independent; programming language: Rust; license: MIT. Audio demos are available as additional files.

## Abbreviations

SPL: Sound pressure level
FIR: Finite impulse response
HV: Heavy vehicle
RPM: Revolutions per minute
LS: Lower source
HS: Higher source
BL: Baseline model
HM: Harmonoise model
HM+BD: Harmonoise + Baldan model
PSD: Power spectral density
FFT: Fast Fourier transform
RMSE: Root mean squared error
MAE: Mean absolute error
SMS: Spectral modeling synthesis

## Footnotes

1 https://www.youtube.com/watch?v=_XbKWmKjdr4

2 https://www.youtube.com/watch?v=fQPnLlXvG9I
